# Stress-Induced Directed Self-Assembly of Perpendicularly Oriented Block Copolymer Lamellae for Lithographic Density Multiplication

**DOI:** 10.1021/acsami.5c14351

**Published:** 2025-09-29

**Authors:** Aum Sagar Panda, Cheng-Hsun Tung, Jui-Chang Chuang, The Anh Nguyen, Thuy Trinh, Pin-Chia Chen, Thanmayee Shastry, Fu-Rong Chen, Ming-Chang Lee, Chang-Chun Lee, Rong-Ming Ho

**Affiliations:** † Department of Chemical Engineering, 34881 National Tsing Hua University, Hsinchu 30013, Taiwan; ‡ Department of Power Mechanical Engineering, National Tsing Hua University, Hsinchu 30013, Taiwan; § Department of Electrical Engineering, National Tsing Hua University, Hsinchu 30013, Taiwan; ∥ Department of Engineering and System Science, National Tsing Hua University, Hsinchu 30013, Taiwan; ⊥ Department of Materials Science and Engineering, City University of Hong Kong, Tat Chee Avenue, Kowloon, Hong Kong 852, China

**Keywords:** block copolymer, vacuum, controlled orientation, finite element analysis, directed self-assembly

## Abstract

This work demonstrates a stress-induced directed self-assembly (DSA) approach to produce unidirectionally oriented perpendicular lamellae in block copolymer (BCP) thin films, achieving a smaller line width and a higher aspect-ratio for pattern transfer with lithographic density multiplication. A free-standing polystyrene-*block*-polydimethylsiloxane (PS-*b*-PDMS) thin film on a transmission electron microscopy (TEM) grid is thermally annealed under high vacuum inside an in situ, temperature-resolved TEM instrument. The high vacuum reduces the surface tension discrepancy between PS and PDMS at high temperatures, creating neutral surfaces at both top and bottom sides of the thin film and facilitating the formation of film-spanning perpendicular lamellae via self-alignment during thermal annealing. Finite element analysis reveals that the *x*-directional stress is concentrated at the grid edge, inducing the formation of unidirectionally oriented perpendicular lamellae, as evidenced by an in situ, time-resolved TEM observation. This edge-parallel alignment arises from a tensile stress gradient along the edge-normal direction, which favors lamellae aligned parallel to the edge to minimize elastic mismatch between PS and PDMS during self-assembly. For nanopatterning, the free-standing thin film is transferred onto substrates with e-beam-defined trenches followed by thermal annealing in a homemade vacuum oven. The BCP film gradually flows into the trenches during which the stress guides the formation of unidirectionally oriented perpendicular lamellae. Subsequently, well-defined SiO_2_ line patterns can be formed within the trenches after O_2_ reactive ion etching. This facile method enables controlled orientation of a free-standing BCP thin film by integrating vacuum-driven perpendicular orientation and stress-induced DSA, providing appealing potential for fabrication of highly ordered line patterns in advanced lithographic applications.

## Graphical abstract

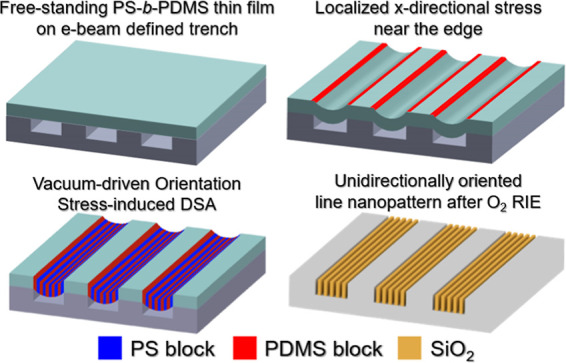

## Introduction

Recent developments in the semiconductor industry have put block copolymers (BCPs) at the forefront of lithography and patterning due to their ability to self-assemble into a variety of well-ordered nanostructures such as a body-centered sphere, hexagonally packed cylinder, and lamellae via microphase separation.^1–3^ Although continual device miniaturization has increasingly relied on top-down extreme ultraviolet lithography (EUV), significant challenges remain in addressing stochastic issues such as critical dimension nonuniformity and line edge roughness, achieving high-fidelity pattern transfer as well as developing new photoresists compatible with new high numerical aperture (high-NA) EUV systems. An alternative patterning strategy is to integrate top-down with bottom-up approaches such as directed self-assembly (DSA) of BCPs.^4–10^ DSA, a concept extensively studied over the past two decades, is now being re-evaluated as a promising strategy to address the scaling and resolution limitations of EUV lithography. Furthermore, DSA has been employed in the nanofabrication of a broad spectrum of nanoscale devices such as graphene nanoribbons,^11–13^ silicon field-effect transistors,^14,15^ nano sensors,^16,17^ nanocatalysts,^18^ and nanoplasmonics.^19^

As device critical dimensions shrink into the sub-10 nm regime, BCPs must combine a high Flory–Huggins segregation strength (χ) with a low degree of polymerization (N) to achieve the necessary domain spacing.^20^ Silicon-containing BCPs such as polystyrene-*b*-polydimethylsiloxane (PS-*b*-PDMS) meet these criteria and possess high etching contrast for efficient pattern transfer, providing a promising platform for next-generation BCP lithography.^21–24^ However, the extremely low surface energy of silicon-containing blocks drives them to wet the air surface, resulting in lamellar or cylindrical nanostructures with a parallel orientation. This preferential wetting results in a major challenge to creating nanostructures perpendicular to the substrate, which are essential for high-fidelity pattern transfer. Many approaches have been developed to achieve perpendicular nanostructures in BCP thin films such as solvent evaporation,^25–27^ substrate modification,^28,29^ shear fields,^30,31^ electric fields,^32,33^ tailored top-coat materials,^34,35^ and plasma surface treatments.^36,37^ While those methods have demonstrated effectiveness in controlling domain orientation, they often involve the use of solvent, additional processing steps, customized materials, or specialized tools, thereby increasing fabrication complexity and cost. These considerations can limit their scalability and compatibility with high-throughput industrial processes.

A recent in situ transmission electron microscopy (TEM) study demonstrated that a free-standing cylinder-forming BCP thin film can achieve a film-spanning perpendicular cylinder under high-vacuum thermal annealing. The perpendicular cylinder emerges because surface energies of constituted homopolymers converge in a high-vacuum and high-temperature environment, giving a neutral surface that drives the perpendicular orientation of the cylinder as observed by time-resolved experiments.^38,39^ Note that this method simply relies on a vacuum chamber equipped with a heating system that is already ubiquitous across semiconductor processing tools, making it well-suited for integration into existing semiconductor manufacturing processes.

To obtain line-and-space patterns suitable for lithographic applications, controlling the lateral order of perpendicular lamellar structures across a large area (i.e., forming unidirectionally perpendicular lamellae) is indispensable. Cylinder-forming BCPs spontaneously self-assemble into hexagonally packed arrays with a locally high order, but the presence of grain boundaries prevents long-range lateral order. By contrast, lamellae-forming BCPs tend to form irregular fingerprint patterns, even when a perpendicular orientation can be achieved. Long-range alignment of line and space patterns has been achieved either by graphoepitaxy,^22,40–44^ where the geometric confinement directs lamellae or a monolayer of a cylinder by preferential wetting of one block toward the sidewall of topographic patterns, or by chemoepitaxy,^43,45–47^ which employs chemical patterns with alternating preferential and neutral stripes to control lamellae forming along a single axis. Another significant advantage of DSA lies in the ability to achieve density multiplication, in which the number of line and space features formed within a given area is greater than that of the guiding templates.^48–50^ The guiding topographic or chemical patterns defined by EUV or electron beam lithography can have a relatively wide pitch length while still directing the formation of much denser nanopatterns. As a result, DSA enables the formation of high-density patterns from low-density templates, overcoming the pattern density limits of conventional lithographic techniques. Note that both DSA approaches still require an underlying layer (usually grafting a random copolymer) to neutralize the interfacial affinity for the two blocks and suppress preferential wetting. By contrast, a free-standing BCP film bound by vacuum on both sides provides inherently symmetric neutral conditions to achieve a film-spanning perpendicular structure, eliminating the need for the preparation of a neutral layer and thus simplifying the whole process for controlled orientation. Furthermore, the mechanical tensile stress localized at the edges of free-standing films may exert additional directional forces on the BCP, potentially contributing to the alignment of the perpendicular lamellae, and thus enhancing unidirectional ordering.^51,52^

Herein, this work demonstrates the stress-induced DSA of a free-standing PS-*b*-PDMS thin film under high-vacuum thermal annealing to create unidirectionally oriented perpendicular lamellae. A PS-*b*-PDMS thin film spin-coated on a Si wafer can be transferred to a TEM grid with a pierced viewing hole. The region of the film suspended is at a free-standing state with no substrate contact, thereby providing symmetric boundary conditions. Under high vacuum, the neutral surfaces at both sides of the thin film promote the formation of film-spanning perpendicular lamellae. Furthermore, this configuration enables in situ, temperature-resolved TEM to observe morphological evolution during high-vacuum thermal annealing. Interestingly, the lamellae were observed to grow progressively from the edge toward the center of the free-standing region with edge-parallel alignment. This unidirectional growth suggests a potential influence of edge-localized tensile stress induced by gravity-driven tension, which guides lamellar alignment and propagation over time. Building on these insights, the film can also be applied to topographically patterned substrates defined by e-beam lithography, where similar tensile stress-induced alignment can be observed after high-vacuum thermal annealing in a vacuum oven. Remarkably, the aligned lamellae can be found to fill trenches, enabling the formation of unidirectionally oriented perpendicular lamellae within confined geometries. Subsequently, an oxygen reactive ion etching (O_2_ RIE) is utilized to selectively etch PS microdomain and simultaneously oxidize PDMS into SiO_2_, giving well-defined SiO_2_ line patterns with long-range lateral order with lithographic density multiplication. This result demonstrates a novel strategy for achieving well-aligned line patterns based on the new concept of stress-induced DSA, offering new opportunities for BCP lithography in advanced semiconductor and nano-MEMS device applications.

## Results and Discussion

### Formation of Perpendicular Lamellae by Thermal Annealing under Vacuum

The creation of unidirectionally oriented perpendicular lamellae involves two simultaneous processes: the formation of perpendicular lamellae via self-alignment of induced perpendicular lamellae from the top and bottom surfaces of the thin film, resulting in film-spanning lamellae, and the alignment of the lamellar normal in a unidirectional orientation to achieve long-range lateral order. Owing to the notorious low surface energy of the PDMS block, the PS-*b*-PDMS thin film tends to form a parallel orientation induced by PDMS surface wetting. Our previous study found that the issue can be overcome by thermal annealing under high vacuum (10^–4^ Pa) at a reasonably high temperature; it is possible to reduce the surface tension discrepancy between PS and PDMS, leading to the formation of a neutral surface that induces perpendicular nanostructures.^38^ Yet, in contrast to cylinders, lamellae do not exhibit long-range directional alignment after thermal annealing under vacuum. Although cylinders may form locally ordered grains, both morphologies require DSA strategies to achieve global orientation and long-range lateral order. Figure S1 schematically illustrates the two-step mechanism of vacuum-driven neutralization, followed by edge-localized tensile-stress alignment. In the first stage, perpendicular lamellae nucleate near the film edge, initiated from both the top and bottom surfaces (Figure S1a). The localized tensile stress at the edges then promotes alignment of the lamellar normal in a unidirectional orientation, while additional self-alignment between the top and bottom lamellae may occur at this stage, giving the film-spanning perpendicular domains (Figure S1b). Finally, the aligned perpendicular lamellae propagate inward and fill the trench, resulting in unidirectionally ordered structures across the entire region (Figure S1c).

### Formation of Unidirectional Perpendicular Lamellae via Stress-Induced DSA

To achieve a free-standing configuration of the BCP thin film, PS-*b*-PDMS was first spin-coated onto a silicon wafer with a native oxide layer to form a thin film with a thickness of approximately 250 nm. The native oxide was then partially etched using hydrofluoric acid (HF), allowing the film to detach and float on the water surface. The film released was subsequently transferred onto substrates designed to support the free-standing condition, giving the neutral surface for the formation of perpendicular lamellae from the top and bottom of the thin film. The sample was collected and then placed on a heating chip with a pierced viewing hole for in situ TEM experiments. Details with respect to the fabrication of such chips can be found in our previous work.^39^ In situ, time-resolved TEM observation was employed to monitor the morphological evolution of the free-standing, lamellae-forming BCP thin film under high-vacuum thermal annealing. Figure 1 shows a series of TEM snapshots of the free-standing PS-*b*-PDMS thin film after thermal annealing at 300 °C under high vacuum (approximately 10^–5^ Pa, vacuum level of TEM) for different annealing times. As shown in Figure 1a, after 5 min of thermal annealing, the nucleation and growth of unidirectionally oriented perpendicular lamellae are initiated from the edge of the TEM grid. By increasing the annealing time to 30 min (Figure 1b), the well-aligned unidirectionally oriented perpendicular lamellae form within a significant area near the grid edge, extending outward from the edge for approximately 500 nm. As shown in Figure 1c, after annealing for 1 h, the perpendicular lamellae grow away from the edge for more than 1 μm at which dislocation defects within the lamellae become evident (inset, Figure 1c). Finally, by increasing the annealing time to 2 h, well-ordered unidirectionally oriented perpendicular lamellae can be observed over a larger area (Figure 1d), demonstrating the suggested nucleation and growth mechanism of unidirectionally oriented perpendicular lamellae from the edge of the TEM grid.

**Figure 1:**
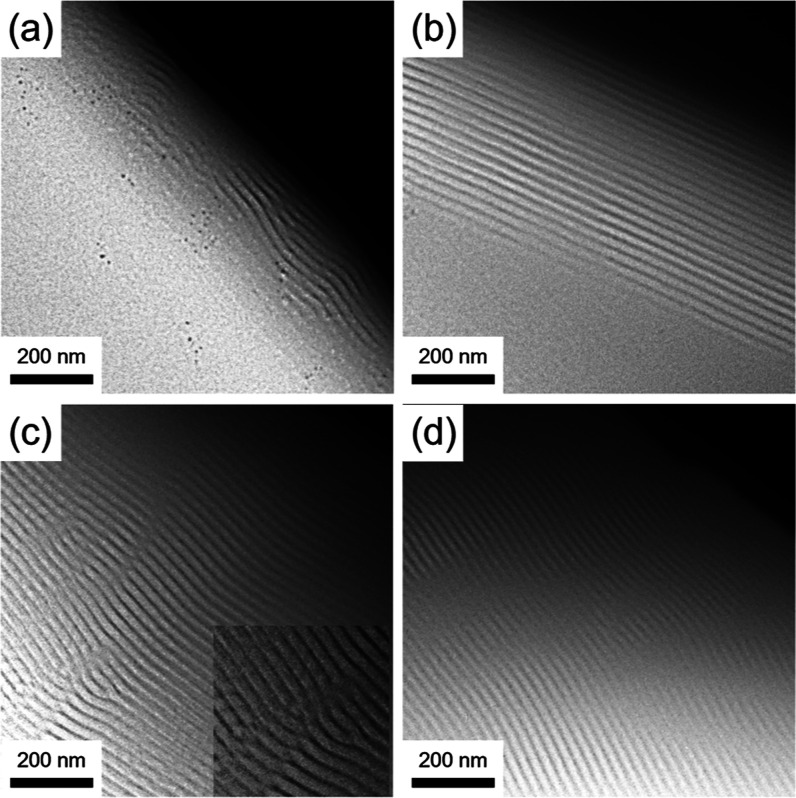
Stress-induced unidirectionally oriented perpendicular lamellae. Time series of in situ, temperature-resolved TEM imaging of a free-standing PS-*b*-PDMS thin film after thermal annealing at 300 °C for (a) 5 min; (b) 30 min; (c) 60 min (the inset shows the defect annihilation process); (d) 120 min.

Yet, it is noted that there is also the occurrence of perpendicular lamellae in the region far away from the edge but with an irregular texture (Figure S2, Supporting Information). We speculate that it might be attributed to the reduction of stress concentration in regions farther from the grid edge at which the influence of tensile stress induced by gravity on the thin film becomes insignificant. Near the edge, unidirectionally oriented perpendicular lamellae can nucleate simultaneously from both the top and bottom surfaces of the free-standing film, facilitating rapid self-alignment due to the consistent orientation. In contrast, farther from the edge, the absence of stress-induced DSA leads to mismatched lamellar orientations at the top and bottom surfaces; as a result, it requires a longer time to execute the self-alignment process. It is reasonable to expect that with the extended annealing time, the unidirectionally perpendicular lamellae near the edge can gradually merge with those irregularly textured lamellae in the interior regions.

Interestingly, the edge dislocations as shown in the inset of Figure 1c can be effectively annihilated with prolonged annealing, thus improving the lateral ordering. The process of defect annihilation in lamellae-forming BCP thin films proceeds through a thermally activated molecular mechanism involving the formation and growth of bridging structures. Those molecular bridges originate from one dislocation core and extend toward neighboring lamellae, ultimately connecting misaligned domains across the defective region.^53,54^ With further thermal annealing (Figure 1d), the formerly defective region becomes incorporated into the unidirectionally oriented perpendicular lamellae with a long-range order.

### Stress Analysis of Free-Standing PS-*b*-PDMS Thin Films

To further investigate the behaviors of stress-induced DSA of the free-standing PS-*b*-PDMS thin film, a theoretical study of stress distribution in the PS-*b*-PDMS thin film induced by gravity was carried out through simulation using finite element analysis (FEA). The effective Young’s modulus of the PS-*b*-PDMS thin film was evaluated by considering its orthotropic material properties based on the constituent moduli of 3.0 GPa for polystyrene (*f*_PS_^v^ = 0.61) and 500 kPa for polydimethylsiloxane (*f*_PDMS_^v^ = 0.39). A 250 nm thick PS-*b*-PDMS thin film was placed on the topographic substrate and configured under different geometrical conditions to study the mechanical behavior of thin films under gravity. As shown in Figure S3a (Supporting Information), L1 is fixed at 250 nm (the film thickness of PS-*b*-PDMS) while L2–L4 are varied across different cases (see Table S1 for details, Supporting Information). The geometry of the copper grid structure was modeled to assess the contribution of mechanical behaviors due to gravity in the free-standing PS-*b*-PDMS thin film on a TEM copper grid (Figure S3b, Supporting Information). The simulation results show that the maximum values of total deformation and *Z*-directional displacement are identical, indicating that the overall deformation is predominantly concentrated in the out-of-plane (*Z*) direction. This results in a concave curvature that spans between adjacent copper grid supports (Figure S3c,d, Supporting Information). The deformation profile suggests a gradual flow of the material toward the bottom, in line with the direction of the gravitational force. As the film flows downward, mechanical tension is generated near the grid edges, at which the material is anchored to the copper grid. The resistance to flow at these boundaries induces lateral stretching, giving rise to tensile stress localized near the contact edge of the copper grid. This behavior is quantitatively confirmed by the FEA results; the induced stress in the free-standing thin film will be concentrated near the edge of the TEM grid and gradually decreases with distance from the edge (Figures S3e, Supporting Information). This stress distribution induced by gravity creates a gradient along the *x*-direction, giving an *x*-directional tensile stress applied across the BCP thin film, providing the driving force for the alignment of the BCP microdomains through the stress-induced DSA process. A similar deformation profile (Figure 2a) and stress distribution (Figure 2b) can be observed in simulations using topographic SiO_2_ substrates with gap width of 2000 nm, indicating that edge-localized tensile stress is a general feature of a free-standing film when supported by a substrate with comparable elastic moduli, such as copper (*E* ∼ 130 GPa) and SiO_2_ (*E* ∼ 75 GPa). This stress localization behavior can also be observed across simulations with varying gap widths (Figure S4a,d, Supporting Information), suggesting that the emergence of tensile stress near the edge is intrinsic regardless of specific geometries and trench dimensions.

**Figure 2:**
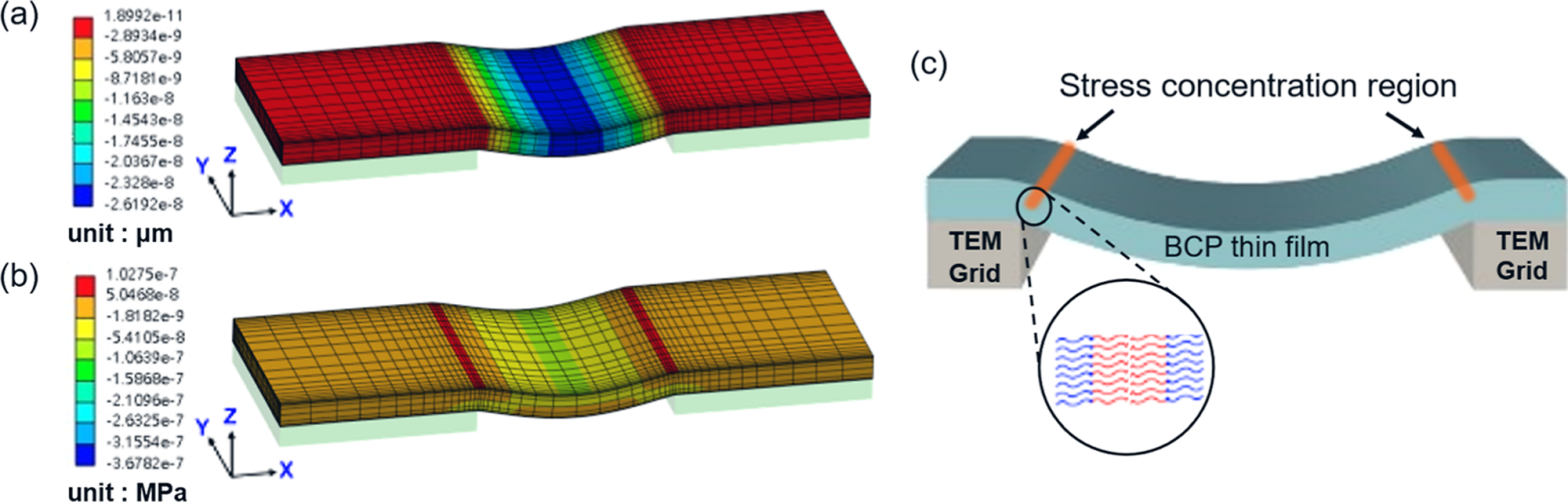
Stress analysis of PS-*b*-PDMS free-standing thin film using FEA analysis. (a) Out-of-plane (*Z*-direction) displacement profile showing maximum deformation at the center of the suspended region. (b) *X*-directional stress distribution of the free-standing PS-*b*-PDMS thin film on the topographically patterned SiO_2_ substrate, with tensile stress localized along the edges of the SiO_2_ mesa (trench widths = 2000 nm). (c) Schematic illustration of stress concentration near the copper bars of a TEM grid, arising from tension induced by film bending under gravity. Nucleation of stress-induced unidirectionally perpendicular lamellae is initiated from the stress concentrated region. The inset shows the preferred orientation of lamellae.

It is reasonable to suggest that the localized stress on the free-standing thin film gives rise to the initiation of the microphase separation, as illustrated in Figure 2c. The formation of unidirectionally oriented perpendicular lamellae along the edge can be correlated with the chain arrangements during self-assembly. Note that there is a high discrepancy in the elastic modulus of constituent blocks (PS and PDMS) that may give rise to an uneven stretching of the PS and PDMS blocks, as shown in Figure S5a (Supporting Information). As a result, the formation of unidirectionally oriented perpendicular lamellae with the lamellar normal perpendicular to the edge is facilitated (Figure S5b, Supporting Information). This suggests that the induced stress at the edge of the TEM grid promotes a self-assembly pathway in which the polymer chains preferentially orient in a manner that minimizes elastic energy, giving rise to edge-parallel, unidirectionally oriented perpendicular lamellae during thermal annealing under vacuum.

### Stress-Induced DSA of a Free-Standing Thin Film on a Topographically Patterned Wafer

As demonstrated above, it is feasible to carry out the stress-induced DSA for the free-standing thin film with unidirectionally oriented perpendicular lamellae. Following the concept, by taking advantage of vacuum-driven and stress-induced DSA for the formation of the unidirectionally oriented perpendicular lamellae with a long-range lateral order, it is feasible to address the PS-*b*-PDMS thin film onto a topographically patterned wafer as illustrated in Figure 3 for film transfer and the corresponding morphological evolution on a topographically patterned wafer. As shown in Figure 3a, a PS-*b*-PDMS thin film with a thickness of approximately 250 nm is prepared on a Si wafer with a native oxide layer through spin coating. After the oxide is etched out with HF, the thin film can be floated on the water surface (Figure 3b) and then transferred to the topographically patterned wafer (Figure 3c), giving a free-standing PS-*b*-PDMS thin film on the patterned wafer (Figure 3d). By taking advantage of vacuum-induced orientation for the free-standing thin film, perpendicular lamellae can be formed from the top and bottom surfaces of the thin film due to the formation of the neutral surface at the air/polymer melt interface at high vacuum conditions (10^–4^ Pa). The thermal annealing is carried out inside a homemade vacuum oven capable of reaching temperatures of up to 400 °C and a high vacuum of 10^–4^ Pa. The detailed design of the vacuum oven can be found in our previous work.^43^ Meanwhile, the free-standing thin film starts flowing (deforming) during thermal annealing (Figure 3e). Owing to the stress-induced DSA as demonstrated above, the formation of the unidirectionally oriented perpendicular lamellae can be initiated from the edge and gradually propagate inward to give the long-range lateral order (Figure 3f). After the film flows into the trench of the topologically patterned wafer, it is possible to successfully fabricate the aimed nanopattern as shown in Figure 3g, giving a well-defined line pattern within the trench for the lithographic applications (see below for details).

**Figure 3:**
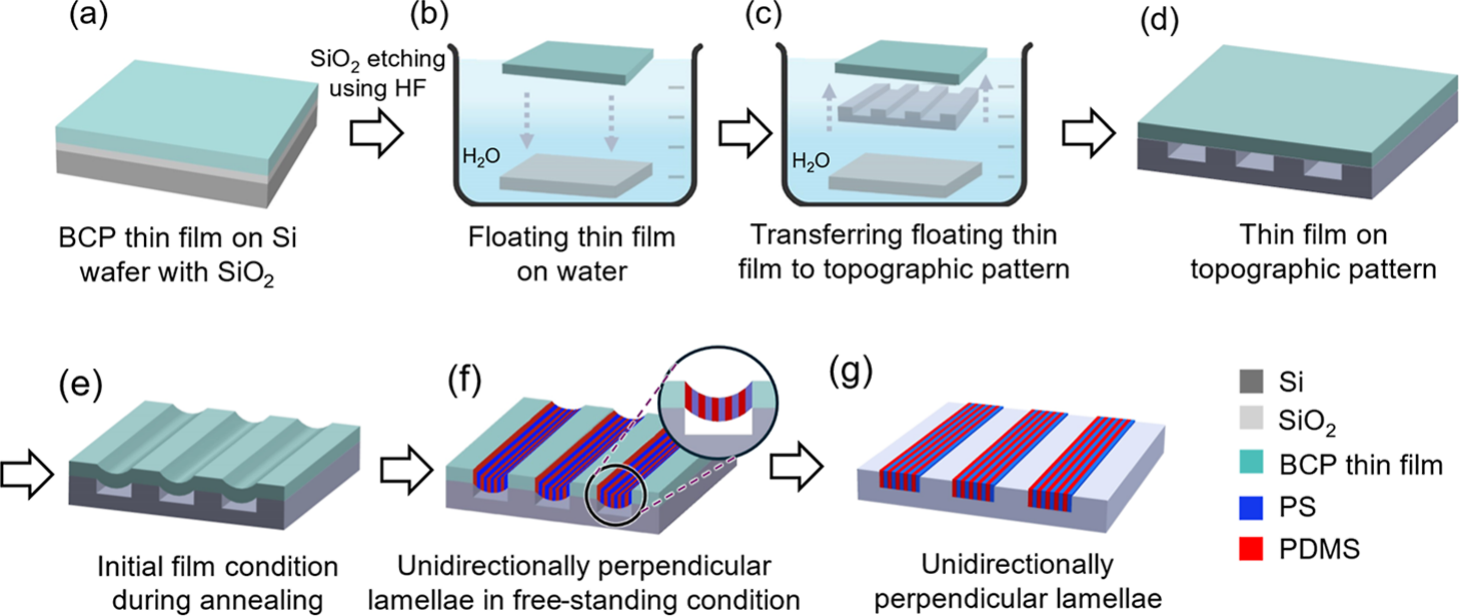
Schematic illustration of the film transfer process and stress-induced DSA on a topographically patterned substrate. (a) A PS-*b*-PDMS thin film on a Si wafer with a native SiO_2_ layer; (b) a floating thin film on water after HF etching of the SiO_2_ layer; (c) transferring the floating thin film to a topographically patterned wafer with e-beam-defined trenches; (d) a free-standing PS-*b*-PDMS thin film on a topographically patterned wafer with e-beam-defined trenches; (e) the initial film conditions under high-vacuum thermal annealing during which the PS-*b*-PDMS thin film flows into the trench; (f) formation of unidirectional perpendicular lamellae in the free-standing thin film; (g) the final film conditions with unidirectionally oriented perpendicular lamellae within the topographic trenches.

### Unidirectionally Oriented Perpendicular Lamellae on a Topographically Pattern Substrate

Figure 4a–c shows the cross-sectional FE-SEM images of the step-by-step fabrication process to achieve the unidirectionally oriented perpendicular lamellae on the topographically patterned wafer. E-beam lithography was employed to obtain topographically patterned wafers containing various trenches with trench widths from approximately 300 to 4000 nm. The stepwise fabrication process of a topographic substrate is illustrated in Figure S6 (Supporting Information). Figure 4a represents the topographically patterned wafer with a trench width and height of about 500 and 200 nm, respectively. The inset gives an enlarged image of the patterned substrate for better visualization. The topographically patterned wafer was further characterized by AFM as shown in Figure S7 (Supporting Information), confirming the well-defined topography with a flat bottom surface and a trench depth of approximately 200 nm. As shown in Figure 4b, the PS-*b*-PDMS thin film is addressed onto the topologically patterned wafer after the film transfer procedure as described in Figure 3, creating a free-standing PS-*b*-PDMS thin film on a patterned wafer. It can be clearly observed that the top and bottom surfaces of the free-standing PS-*b*-PDMS thin film are exposed to air; as a result, during thermal annealing under high vacuum, both the top and bottom surfaces of the thin film can induce perpendicular lamellae owing to the formation of a neutral surface. As shown in Figure S8a, films suspended across 500 nm trenches exhibit a concave profile after a short thermal annealing time, while in wider trenches of 3 μm (Figure S8b), the gravitational deformation becomes more pronounced. These observations confirm that gravitational deformation occurs under the annealing conditions, consistent with the FEA simulation. As expected, after thermal annealing, the PS-*b*-PDMS thin films will flow into the trench (Figure 4c), and the aimed unidirectionally oriented perpendicular lamellae with long-range lateral order emerge in this stage. To evidence the aimed texture, an O_2_ RIE was performed to create the SiO_2_ line pattern by removing the PS microdomain and simultaneously oxidizing the PDMS cylindrical microdomain into SiO_2_; as shown in the inset of Figure 4c, well-defined perpendicular lamellae can be clearly identified within a 300 nm-wide trench, confirming the successful lamellae alignment from the stress-induced DSA. The corresponding top-view FE-SEM image (Figure 4d) further demonstrates the perfect alignment of the SiO_2_ line pattern within a 500 nm-wide trench. Moreover, the lamellar pitch length can be measured as approximately 31 nm by fast Fourier transfer from the top-view micrograph (see the inset of Figure 4d). To highlight the importance of thermal annealing under high vacuum, a comparative experiment was conducted in which a free-standing PS-*b*-PDMS thin film was thermally annealed under ambient pressure. As shown in Figure S9 (Supporting Information), only parallel lamellae formed within the trench after thermal annealing under ambient pressure (10^5^ Pa), indicating that a vacuum-driven orientation is essential for achieving the desired perpendicular orientation. This result also suggests that stress alone is insufficient to induce the formation of perpendicular lamellae within the trench. In contrast, high-vacuum conditions (10^–4^ Pa) are indispensable, as they enable the formation of neutral surfaces that are essential for creating the perpendicular nanostructure.

**Figure 4:**
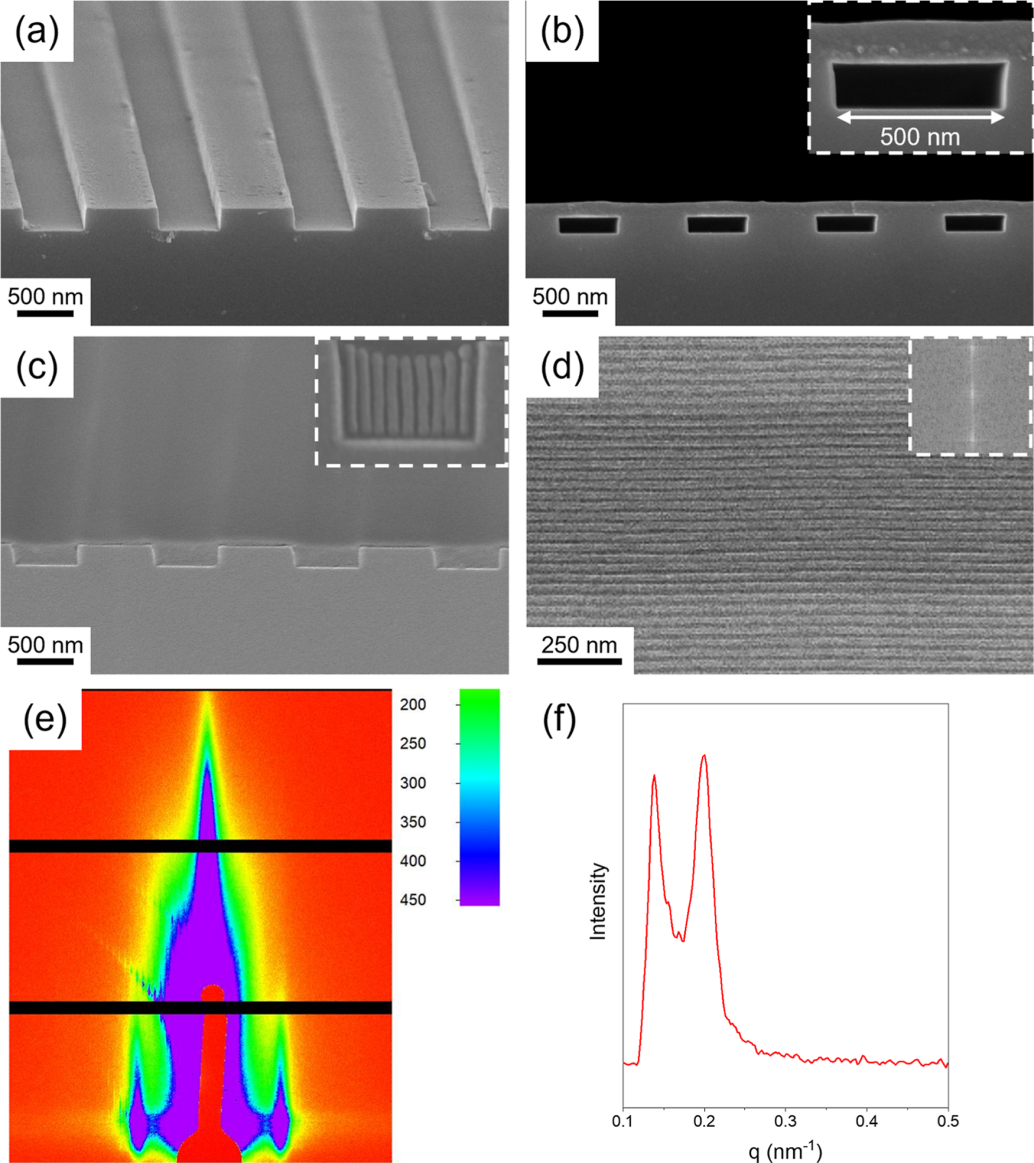
Cross-sectional FE-SEM images of (a) a topographically patterned wafer with trenches; (b) a free-standing PS-*b*-PDMS thin film on the topographic trench; (c) PS-*b*-PDMS thin film conformally filling the topographic trench after thermal annealing under vacuum. The inset shows the unidirectionally oriented perpendicular lamellae within the trench after O_2_ RIE; (d) a top-view FE-SEM image of unidirectionally oriented perpendicular lamellae after O_2_ RIE; (e) a 2D GISAXS pattern of unidirectionally oriented perpendicular lamellae after O_2_ RIE; (f) the 1D integral profile of GISAXS.

The long-range order of the SiO_2_ line pattern was investigated using GISAXS at an incident angle of 0.2°. Figure 4e shows the corresponding 2D GISAXS patterns, in which a sharp scattering signal appears prominently below the Yoneda band, confirming the formation of well-ordered lamellar structures from the whole film thickness rather than surface scattering. Notably, there is no detectable scattering peak along the *q*_*z*_ axis, indicating the absence of lamellae parallel to the substrate. This result supports the observed results that the lamellae are predominantly oriented perpendicularly to the substrate due to the vacuum-induced perpendicular orientation nucleated from both the top and bottom surfaces of the thin film. Furthermore, the corresponding 1D GISAXS integration in Figure 4f reveals a primary scattering peak (indicated by the red arrow) at *q* ∼ 0.2 nm^–1^, corresponding to a lamellar pitch length of approximately 31.4 nm, further validating the uniformity and precise periodicity of the resulting SiO_2_ line pattern with the long-range order as suggested. Furthermore, the azimuthal peak exhibited a fwhm of Δ*q* = 0.02345 nm^–1^, corresponding to a coherence length of approximately 240–270 nm depending on the peak-shape model (Gaussian or Lorentzian). This value reflects the average correlation length of ordered domains over the illuminated area and is sensitive to orientation distributions and minor defects across the trenches. Notably, SEM images (Figure 4d) show that lamellae can span across the entire 500 nm trench, indicating that local grains can extend beyond the average coherence length obtained from GISAXS.

Most importantly, the results presented above demonstrate the feasibility of density multiplication. Note that the pitch length of the trench pattern refers to the full pitch, including both the mesa width and the trench width. In the e-beam layout design, the mesa and trench are defined with a 1:1 width ratio, resulting in a trench pattern pitch equal to twice the trench width. In the case of a 300 nm-wide trench, nine layers of line patterns with a pitch length of approximately 31.4 nm can be generated within the trench (inset of Figure 4c) after O_2_ RIE treatment. This corresponds to a density multiplication factor of 18× relative to the original trench pattern pitch of 600 nm defined by e-beam lithography. As a result, a low-density guiding pattern can effectively direct the formation of a high-density lamellar nanostructure.

Figure 5 shows the FE-SEM images of unidirectionally oriented perpendicular lamellae in different trench widths; free-standing PS-*b*-PDMS thin films on patterned wafers were thermally annealed under high vacuum at 300 °C for 2 h followed by O_2_ RIE to create the SiO_2_ line pattern. In contrast to the unidirectionally oriented perpendicular lamellae acquired using the TEM grid, the forming unidirectionally oriented perpendicular lamellae on topographically patterned wafers clearly give better control on lateral ordering regardless of the trench widths examined. Note that the gap width of the TEM grid is much greater than the trench width of the topographical patterns, resulting in a diminished effect of the stress at the edges. In patterned trenches, the proximity of the opposing sidewalls enables stress fields to propagate from both edges, guiding the lamellae to a more uniform lateral orientation. Note that initial conditions such as trench width are critical for the aimed induced stress in a free-standing PS-*b*-PDMS thin film. Interestingly, the aimed unidirectionally oriented perpendicular lamellae can be successfully achieved by using trench widths ranging from 300 to 1000 nm. Figure 5a shows the formation of unidirectionally oriented perpendicular lamellae with a trench width of 300 nm. Despite slight pitch variations near the edge, possibly caused by limited polymer mobility and a bowl-shaped thickness profile during thermal flow, it can be observed that the lateral ordering of the unidirectionally oriented perpendicular lamellae has significant improvement as compared to the use of TEM grid. Those imperfections can be effectively annihilated by increasing the trench widths beyond 300 nm. As shown in Figure 5b,c, when the trench widths are increased to 500 and 750 nm, respectively, well-ordered, unidirectionally oriented perpendicular lamellae with a uniform pitch length can be observed with the annihilation of uneven spacing caused by the narrower trench width. Interestingly, the formation of unidirectionally oriented perpendicular lamellae can be observed on the mesa regions; it might be attributed to the vacuum-driven orientation of perpendicular lamellae from the top and stress-induced lateral alignment remains effective in these regions even though the substrate effect might cause the formation of parallel lamellae. Note that the perpendicular lamellae nucleated near the trench edge can propagate not only inward into the trench but also outward onto the mesa, consistent with the *x*-directional stress gradient revealed in the FEA analysis (Figure 2b), which extends both inward and outward from the trench edge. Figure 5d represents the SiO_2_ line patterns in a wider trench (width ∼1000 nm), showing the feasibility of the suggested stress-induced DSA approach even in a micrometer scale trench width.

**Figure 5:**
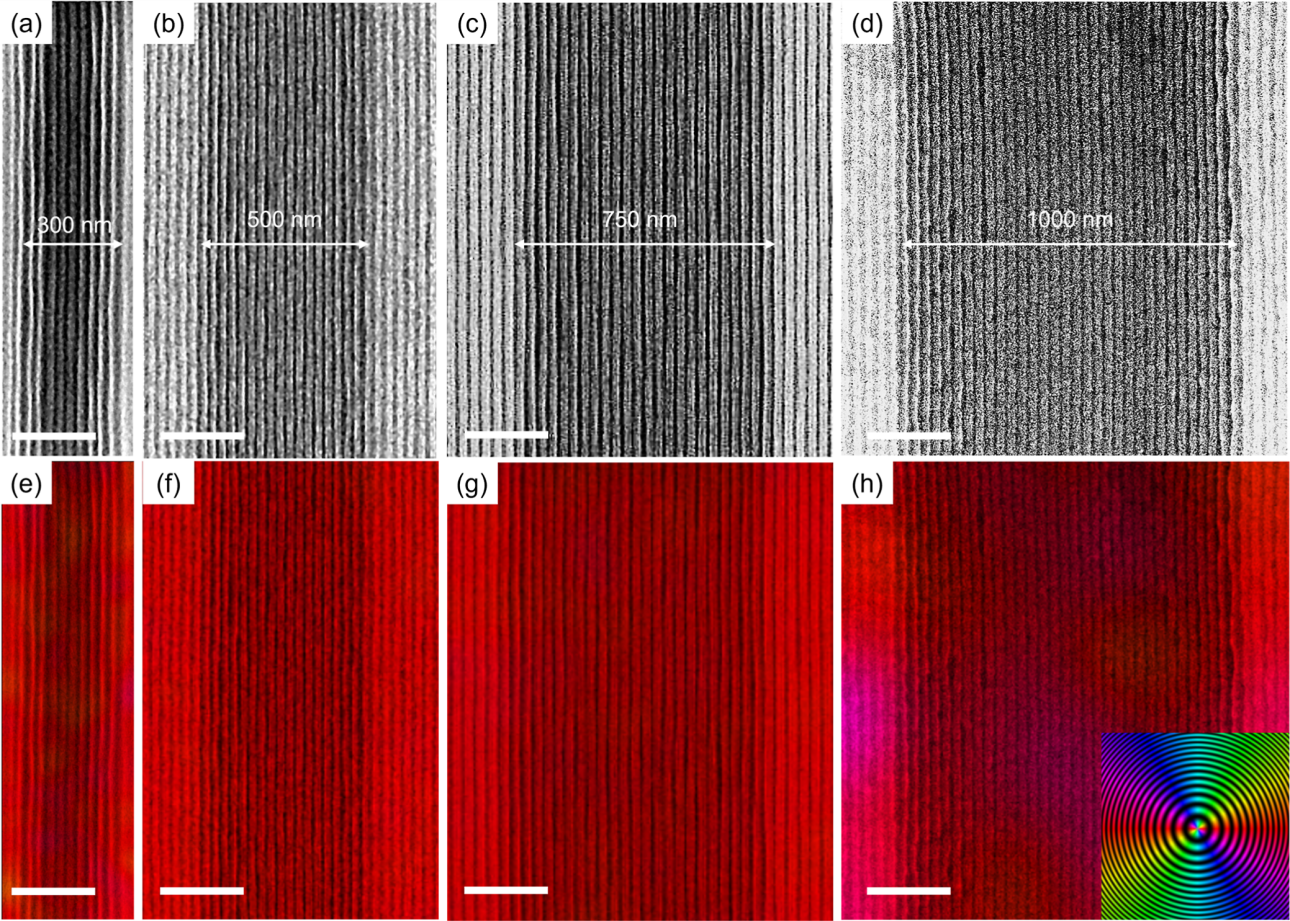
FE-SEM images of well-aligned SiO_2_ line patterns from the free-standing PS-*b*-PDMS thin film on a topographic pattern with trench widths of (a) 300 nm; (b) 500 nm; (c) 750 nm; (d) 1000 nm. The thin films were thermally annealed under vacuum (10^–4^ Pa) at 300 °C for 2 h followed by O_2_ RIE (the scale bars are 250 nm). The lamellar nanostructures exhibit unidirectional orientation with increasing domain density as the trench width increases, corresponding to density multiplication factors of approximately (a) 18×, (b) 34×, (c) 50×, and (d) 66×, respectively, relative to the trench pitch length defined by e-beam lithography. (e–h) Orientational color analysis of well-aligned SiO_2_ line patterns from the free-standing PS-*b*-PDMS thin film on a topographic pattern with trench widths of (e) 300 nm; (f) 500 nm; (g) 750 nm; (h) 1000 nm. The scale bars are 250 nm. The inset shows the orientational color bar.

As shown in the orientational color map (Figure 5e–h), the unidirectional lamellae are represented by a single color, indicating a high degree of uniformity in the orientation. Notably, the multiplication factor increases proportionally with the trench width. Trench widths of 300, 500, 750, and 1000 nm yield approximate multiplication factors of 18, 34, 50, and 66×, respectively, relative to the pitch lengths of trench patterns, demonstrating the scalability of this approach for achieving density multiplication. However, it is difficult to form the unidirectionally oriented perpendicular lamellae beyond the trench width of ∼1000 nm on a reasonable time scale. It might be attributed to the required long time for the merging of the perpendicular lamellae from both edges of a trench, and a wider trench width might cause the bending of the free-standing thin film to reach the substrate before the occurrence of merging of the perpendicular lamellae. Accordingly, there should be a range of optimized trench widths to give the aimed orientation from stress-induced DSA.

## Conclusion

This work successfully demonstrates the fabrication of free-standing PS-*b*-PDMS thin films on patterned wafers to achieve unidirectionally oriented perpendicular lamellae through stress-induced orientation by thermal annealing under high vacuum, giving a smaller line definition and higher aspect-ratio for pattern transfer with lithographic density multiplication. Owing to the free-standing condition of PS-*b*-PDMS thin film for thermal annealing under a high vacuum (10^–4^ Pa), perpendicularly oriented lamellae can be formed due to the formation of neutral surfaces at the air/polymer melt interfaces. Most importantly, the aimed unidirectional perpendicular lamellae can be achieved due to stress on the free-standing thin film induced by gravity with the use of topographically patterned wafers for DSA to create the long-range lateral order of the forming perpendicular lamellae. Subsequently, a well-aligned SiO_2_ line pattern can be fabricated by using the unidirectional perpendicular lamellae for the O_2_ RIE treatment, which can serve as a lithographic mask for pattern transfer to functional materials with density multiplication for lithographic applications. In summary, the combination of vacuum-driven and stress-induced DSA on topographically patterned wafers enables the fabrication of well-defined line patterns within the trenches, giving appealing platforms with the combination of top-down and bottom-up methods for semiconductor devices and nano-MEMS applications.

## Methods

### Material Synthesis

The synthetic details of the PS-*b*-PDMS diblock copolymer was reported in our previous work,^38,55^ involving a sequential anionic polymerization technique incorporating styrene and hexamethylcyclotrisiloxane. This process employed *sec*-BuLi as an initiator and trimethylchlorosilane (CH_3_)_3_SiCl as a termination reagent under high vacuum conditions. Consequently, a lamella-forming PS-*b*-PDMS with the volume fraction of PDMS, *f*_PDMS_^v^ = 0.39 (*M*_n,PS_ = 14,000 g mol^–1^, *M*_n,PDMS_ = 9000 g mol^–1^, *D̵* = 1.03), was achieved.

### Sample Preparation

PS-*b*-PDMS thin films with a thickness of approximately 250 nm were prepared through spin-coating of dissolved PS-*b*-PDMS (3 wt % in cyclohexane) onto a silicon wafer with a native silicon oxide layer. Free-standing thin films were prepared by delicately removing the silicon oxide layer from the wafer by using a HF aqueous solution, followed by transferring the films onto water and subsequently onto a TEM grid or topographically patterned wafers.

### In Situ TEM Observation

A pulse-heating holder equipped with a 17.5 μm-wide platinum spiral heater (with a 10 nm Cr adhesion layer and 250 nm Si_3_N_4_ encapsulation) suspends the BCP sample. Four-point contacts allow simultaneous heating and resistance-based temperature sensing. Details of the fabrication of microheating chip, electron dosage control, and chip calibration procedures are provided in our previous report.^39^ Bright-field images were captured using a JEM-2100 (JEOL, Ltd.) instrument equipped with the pole piece of a high-contrast objective lens and LaB_6_ thermionic electron emission source. The basic setup of the in situ heating system in TEM includes a wire-connected micro heater, heating holder, and power supply (Keithley 2635B) with an electric power of 3 mW to program the controlled temperature for in situ observation of the ordering process of the lamellae-forming PS-*b*-PDMS thin films under TEM.

### E-Beam Lithography

The topographically patterned silicon wafers for stress-induced DSA were fabricated by using e-beam lithography. The process began with growing a silicon dioxide layer of approximately 200 nm on the silicon substrate, which served as a hard mask. Further, the wafer was spin-coated with e-beam chemically amplified positive tone photoresist (TDUR-P015), followed by e-beam lithography to write submicrometer trench patterns on the photoresist layer. After the exposure, the wafer underwent a development step to reveal the e-beam-patterned photoresist. The final step involved transferring these patterns onto the underlying silicon dioxide hard mask through an inductively coupled plasma etcher. SiO_2_ was etched using CF_4_ to obtain the desired trench features as defined by the e-beam lithography.

### Reactive Ion Etching

Thin-film samples were treated with RIE using O_2_ as an etchant to generate a topographic contrast for subsequent FE-SEM imaging. RIE treatments were carried out on a cello technology, model Nasca-20, operating with 13.56 MHz RF source and maximum RIE power at 300 W. The O_2_ RIE was conducted at an RF power of 100 W, pressure of 60 mTorr, and gas flow rate of 10 sccm. For top-view SEM imaging, the samples were etched for 30 s to reveal the surface morphology. In contrast, a longer etching duration of 200 s was employed to achieve sufficient etched depth and reveal the height contrast of the lamellar nanostructures in cross-sectional SEM images.

### Scanning Electron Microscopy

A HITACHI SU-8010 field emission scanning electron microscopy (FE-SEM) instrument was employed, operating at a 10 keV accelerating voltage with an 8 mm working distance. Prior to imaging, the samples were securely mounted onto carbon conductive adhesive tape and then sputter-coated with a thin layer of platinum (approximately 2 nm thickness) to prevent charging effects.

### Grazing-Incidence Small Angle X-ray Scattering

GISAXS experiments were conducted at the BL23A beamline within the National Synchrotron Radiation Research Center (NSRRC) in Taiwan. A monochromatic beam with an energy of 10 kV and a wavelength (λ) of 1.55 Å was employed with the incident angle carefully set at 0.2°. The scattering data were collected using a MAR165 CCD detector, covering the q region ranging from 0.004 to 0.15 Å^–1^.

## Supplementary Material



## Notes

Schematic illustration of a two-step mechanism of stress-induced DSA; TEM micrograph of a free-standing PS-*b*-PDMS thin film away from the grid edge; FEA analysis of free-standing PS-*b*-PDMS thin films on copper grids; tensile stress distribution of free-standing PS-*b*-PDMS thin films on SiO_2_ topographic substrates; schematic illustration of the preferred lamellar orientation; schematic illustration for fabrication of trench patterns; AFM 3D image and line profile of trench patterns; cross-sectional SEM images of PS-*b*-PDMS thin films supported on the trench width after short time thermal annealing; cross-sectional FE-SEM image of parallel lamellae within the trench; and geometric parameters used in the FEA model (PDF)

## References

[1] Bates F. S., Fredrickson G. H. (1990). Block Copolymer Thermodynamics: Theory and Experiment. Annu. Rev. Phys. Chem..

[2] Bates F. S., Fredrickson G. H. (1999). Block Copolymers
Designer Soft Materials. Phys. Today.

[3] Park C., Yoon J., Thomas E. L. (2003). Enabling Nanotechnology with Self
Assembled Block Copolymer Patterns. Polymer.

[4] Hawker C. J., Russell T. P. (2005). Block Copolymer
Lithography: Merging “Bottom-Up”
with “Top-Down” Processes. MRS
Bull..

[5] Segalman R. A. (2005). Patterning
with Block Copolymer Thin Films. Mater. Sci.
Eng., R.

[6] Cheng J. Y., Ross C. A., Smith H. I., Thomas E. L. (2006). Templated Self-Assembly
of Block Copolymers: Top-Down Helps Bottom-Up. Adv. Mater..

[7] Ruiz R., Kang H., Detcheverry F. A., Dobisz E., Kercher D. S., Albrecht T. R., De Pablo J. J., Nealey P. F. (2008). Density Multiplication
and Improved Lithography by Directed Block Copolymer Assembly. Science.

[8] Luo M., Epps III T. H. (2013). Directed Block Copolymer
Thin Film Self-Assembly: Emerging
Trends in Nanopattern Fabrication. Macromolecules.

[9] Hu H., Gopinadhan M., Osuji C. O. (2014). Directed Self-Assembly of Block Copolymers:
A Tutorial Review of Strategies for Enabling Nanotechnology with Soft
Matter. Soft Matter.

[10] Bates C. M., Maher M. J., Janes D. W., Ellison C. J., Willson C. G. (2014). Block Copolymer
Lithography. Macromolecules.

[11] Bai J., Zhong X., Jiang S., Huang Y., Duan X. (2010). Graphene Nanomesh. Nat. Nanotechnol..

[12] Kim B. H., Kim J. Y., Jeong S. J., Hwang J. O., Lee D. H., Shin D. O., Choi S. Y., Kim S. O. (2010). Surface Energy Modification
by Spin-Cast, Large-Area Graphene Film for Block Copolymer Lithography. ACS Nano.

[13] Son J. G., Son M., Moon K. J., Lee B. H., Myoung J. M., Strano M. S., Ham M. H., Ross C. A. (2013). Sub-10 nm Graphene Nanoribbon Array
Field-Effect Transistors Fabricated by Block Copolymer Lithography. Adv. Mater..

[14] Tsai H., Pitera J. W., Miyazoe H., Bangsaruntip S., Engelmann S. U., Liu C. C., Cheng J. Y., Bucchignano J. J., Klaus D. P., Joseph E. A., Sanders D. P., Colburn M. E., Guillorn M. A. (2014). Two-Dimensional Pattern Formation
Using Graphoepitaxy
of PS-b-PMMA Block Copolymers for Advanced FinFET Device and Circuit
Fabrication. ACS Nano.

[15] Liu C. C., Franke E., Mignot Y., Xie R., Yeung C. W., Zhang J., Chi C., Zhang C., Farrell R., Lai K. F., Tsai H., Felix N., Corliss D. (2018). Directed Self-Assembly
of Block Copolymers for 7 Nanometre FinFET Technology and Beyond. Nat. Electron..

[16] Jung Y. S., Jung W., Tuller H. L., Ross C. A. (2008). Nanowire Conductive
Polymer Gas Sensor Patterned Using Self-Assembled Block Copolymer
Lithography. Nano Lett..

[17] Jeong C. K., Jin H. M., Ahn J. H., Park T. J., Yoo H. G., Koo M., Choi Y. K., Kim S. O., Lee K. J. (2014). Electrical Biomolecule
Detection Using Nanopatterned Silicon via Block Copolymer Lithography. Small.

[18] Choi Y., Cha S. K., Ha H., Lee S., Seo H. K., Lee J. Y., Kim H. Y., Kim S. O., Jung W. (2019). Unravelling
Inherent Electrocatalysis of Mixed-Conducting Oxide Activated by Metal
Nanoparticle for Fuel Cell Electrodes. Nat.
Nanotechnol..

[19] Baek K. M., Kim J. M., Jeong J. W., Lee S. Y., Jung Y. S. (2015). Sequentially
Self-Assembled Rings-in-Mesh Nanoplasmonic Arrays for Surface-Enhanced
Raman Spectroscopy. Chem. Mater..

[20] Sinturel C., Bates F. S., Hillmyer M. A. (2015). High χ–Low
N Block Polymers:
How Far Can We Go?. ACS Macro Lett..

[21] Chan V. Z. H., Hoffman J., Lee V. Y., Iatrou H., Avgeropoulos A., Hadjichristidis N., Miller R. D., Thomas E. L. (1999). Ordered Bicontinuous
Nanoporous and Nanorelief Ceramic Films from Self Assembling Polymer
Precursors. Science.

[22] Jung Y. S., Ross C. A. (2007). Orientation-Controlled Self-Assembled
Nanolithography
Using a Polystyrene–Polydimethylsiloxane Block Copolymer. Nano Lett..

[23] Cushen J. D., Otsuka I., Bates C. M., Halila S., Fort S., Rochas C., Easley J. A., Rausch E. L., Thio A., Borsali R., Willson C. G., Ellison C. J. (2012). Oligosaccharide/Silicon-Containing
Block Copolymers with 5 nm Features for Lithographic Applications. ACS Nano.

[24] Lo T. Y., Krishnan M. R., Lu K. Y., Ho R. M. (2018). Silicon-Containing
Block Copolymers for Lithographic Applications. Prog. Polym. Sci..

[25] Kim S. H., Misner M. J., Xu T., Kimura M., Russell T. P. (2004). Highly
Oriented and Ordered Arrays from Block Copolymers via Solvent Evaporation. Adv. Mater..

[26] Ho R. M., Tseng W. H., Fan H. W., Chiang Y. W., Lin C. C., Ko B. T., Huang B. H. (2005). Solvent-Induced
Microdomain Orientation
in Polystyrene-b-Poly­(l-lactide) Diblock Copolymer Thin Films for
Nanopatterning. Polymer.

[27] Albert J. N., Young W. S., Lewis R. L., Bogart T. D., Smith J. R., Epps T. H. (2012). Systematic Study
on the Effect of Solvent Removal Rate on the Morphology of Solvent
Vapor Annealed ABA Triblock Copolymer Thin Films. ACS Nano.

[28] Mansky P., Liu Y., Huang E., Russell T. P., Hawker C. (1997). Controlling Polymer-Surface
Interactions with Random Copolymer Brushes. Science.

[29] She M. S., Lo T. Y., Ho R. M. (2013). Long-Range
Ordering of Block Copolymer
Cylinders Driven by Combining Thermal Annealing and Substrate Functionalization. ACS Nano.

[30] Singh G., Yager K. G., Berry B., Kim H. C., Karim A. (2012). Dynamic Thermal
Field-Induced Gradient Soft-Shear for Highly Oriented Block Copolymer
Thin Films. ACS Nano.

[31] Oh J., Shin M., Kim I. S., Suh H. S., Kim Y., Kim J. K., Bang J., Yeom B., Son J. G. (2021). Shear-Rolling
Process for Unidirectionally and Perpendicularly Oriented Sub-10-nm
Block Copolymer Patterns on the 4 in Scale. ACS Nano.

[32] Morkved T. L., Lu M., Urbas A. M., Ehrichs E. E., Jaeger H. M., Mansky P., Russell T. P. (1996). Local Control
of Microdomain Orientation in Diblock
Copolymer Thin Films with Electric Fields. Science.

[33] Xu T., Zvelindovsky A. V., Sevink G. J. A., Lyakhova K. S., Jinnai H., Russell T. P. (2005). Electric
Field Alignment of Asymmetric Diblock Copolymer
Thin Films. Macromolecules.

[34] Bates C. M., Seshimo T., Maher M. J., Durand W. J., Cushen J. D., Dean L. M., Blachut G., Ellison C. J., Willson C. G. (2012). Polarity-Switching
Top Coats Enable Orientation of Sub–10-nm Block Copolymer Domains. Science.

[35] Kim E., Kim W., Lee K. H., Ross C. A., Son J. G. (2014). A Top Coat with
Solvent Annealing Enables Perpendicular Orientation of Sub-10 nm Microdomains
in Si-Containing Block Copolymer Thin Films. Adv. Funct. Mater..

[36] Lu K. Y., Lo T. Y., Georgopanos P., Avgeropoulos A., Shi A. C., Ho R. M. (2017). Orienting Silicon-Containing
Block
Copolymer Films with Perpendicular Cylinders via Entropy and Surface
Plasma Treatment. Macromolecules.

[37] Oh J., Suh H. S., Ko Y., Nah Y., Lee J. C., Yeom B., Char K., Ross C. A., Son J. G. (2019). Universal
Perpendicular Orientation of Block Copolymer Microdomains Using a
Filtered Plasma. Nat. Commun..

[38] Panda A. S., Lee Y. C., Hung C. J., Liu K. P., Chang C. Y., Manesi G. M., Avgeropoulos A., Tseng F. G., Chen F. R., Ho R. M. (2022). Vacuum-Driven Orientation
of Nanostructured Diblock Copolymer Thin
Films. ACS Nano.

[39] Hung C. J., Panda A. S., Lee Y. C., Liu S. Y., Lin J. W., Wang H. F., Avgeropoulos A., Tseng F. G., Chen F. R., Ho R. M. (2023). Direct Visualization of the Self-Alignment Process for Nanostructured
Block Copolymer Thin Films by Transmission Electron Microscopy. ACS Macro Lett..

[40] Chai J., Wang D., Fan X., Buriak J. M. (2007). Assembly of Aligned
Linear Metallic Patterns on Silicon. Nat. Nanotechnol..

[41] Chai J., Buriak J. M. (2008). Using Cylindrical Domains of Block
Copolymers To Self-Assemble
and Align Metallic Nanowires. ACS Nano.

[42] Bita I., Yang J. K., Jung Y. S., Ross C. A., Thomas E. L., Berggren K. K. (2008). Graphoepitaxy of Self-Assembled Block Copolymers on
Two-Dimensional Periodic Patterned Templates. Science.

[43] Sun Z., Chen Z., Zhang W., Choi J., Huang C., Jeong G., Coughlin E. B., Hsu Y., Yang X., Lee K. Y., Kuo D. S., Xiao S., Russell T. P. (2015). Directed
Self-Assembly of Poly­(2-vinylpyridine)-b-polystyrene-b-poly­(2-vinylpyridine)
Triblock Copolymer with Sub-15 nm Spacing Line Patterns Using a Nanoimprinted
Photoresist Template. Adv. Mater..

[44] Tung C. H., Ye F., Li W. Y., Nguyen T. A., Lee M. C., Wen T., Guo Z. H., Cheng S. Z. D., Ho R. M. (2024). Directed Self-Assembly
of Polystyrene-Block-Polyhedral Oligomeric Silsesquioxane Monolayer
by Nano-Trench for Nanopatterning. Small.

[45] Ouk
Kim S., Solak H. H., Stoykovich M. P., Ferrier N. J., De Pablo J. J., Nealey P. F. (2003). Epitaxial Self-Assembly of Block Copolymers on Lithographically
Defined Nanopatterned Substrates. Nature.

[46] Park S. M., Stoykovich M. P., Ruiz R., Zhang Y., Black C. T., Nealey P. F. (2007). Directed Assembly of Lamellae- Forming
Block Copolymers
by Using Chemically and Topographically Patterned Substrates. Adv. Mater..

[47] Ji S., Wan L., Liu C. C., Nealey P. F. (2016). Directed Self-Assembly of Block Copolymers
on Chemical Patterns: A Platform for Nanofabrication. Prog. Polym. Sci..

[48] Liu C. C., Ramírez-Hernández A., Han E., Craig G. S., Tada Y., Yoshida H., Kang H., Ji S., Gopalan P., De Pablo J. J., Nealey P. F. (2013). Chemical Patterns
for Directed Self-Assembly of Lamellae-Forming Block Copolymers with
Density Multiplication of Features. Macromolecules.

[49] Liu G., Thomas C. S., Craig G. S., Nealey P. F. (2010). Integration of Density
Multiplication in the Formation of Device-Oriented Structures by Directed
Assembly of Block Copolymer–Homopolymer Blends. Adv. Funct. Mater..

[50] Maher M. J., Rettner C. T., Bates C. M., Blachut G., Carlson M. C., Durand W. J., Ellison C. J., Sanders D. P., Cheng J. Y., Willson C. G. (2015). Directed Self-Assembly of Silicon-Containing Block
Copolymer Thin Films. ACS Appl. Mater. Interfaces.

[51] Albalak R. J., Thomas E. L. (1993). Microphase Separation of Block Copolymer Solutions
in a Flow Field. J. Polym. Sci., Part B:Polym.
Phys..

[52] Honeker C. C., Thomas E. L. (1996). Impact of Morphological
Orientation in Determining
Mechanical Properties in Triblock Copolymer Systems. Chem. Mater..

[53] Hur S. M., Thapar V., Ramírez-Hernández A., Khaira G., Segal-Peretz T., Rincon-Delgadillo P. A., Li W., Müller M., Nealey P. F., De Pablo J. J. (2015). Molecular
Pathways for Defect Annihilation in Directed Self-Assembly. Proc. Natl. Acad. Sci. U.S.A..

[54] Hur S. M., Thapar V., Ramírez-Hernández A., Nealey P. F., De Pablo J. J. (2018). Defect Annihilation Pathways in Directed
Assembly of Lamellar Block Copolymer Thin Films. ACS Nano.

[55] Georgopanos P., Lo T. Y., Ho R. M., Avgeropoulos A. (2017). Synthesis,
Molecular Characterization and Self-Assembly of (PS-b-PDMS)­n Type
Linear (n = 1, 2) and Star (n = 3, 4) Block Copolymers. Polym. Chem..

