# Supplementary material for: Stress-Induced Directed Self-Assembly of Perpendicularly Oriented Block Copolymer Lamellae for Lithographic Density Multiplication
Source: ACS Appl Mater Interfaces. 2025 Sep 29;17(40):56542–52. doi: 10.1021/acsami.5c14351 (PMC12516688; doi:10.1021/acsami.5c14351)
Supplement: Supplementary file 1 [file am5c14351_si_001.pdf]

Supplementary Information

Stress-Induced Directed Self-Assembly of Perpendicularly Oriented Block Copolymer Lamellae for Lithographic Density Multiplication

Aum Sagar Panda[1 $], Cheng-Hsun Tung[1 $], Jui-Chang Chuang[2 $], The Anh Nguyen[3], Thuy Trinh[3], Pin-Chia Chen[1], Thanmayee Shastry[1], Fu-Rong Chen[4,5], Ming-Chang Lee[3], Chang-Chun Lee[2*] and Rong-Ming Ho[1*]

[1] Department of Chemical Engineering, National Tsing Hua University, Hsinchu, Taiwan 30013

[2] Department of Power Mechanical Engineering, National Tsing Hua University, Hsinchu, Taiwan 30013

[3] Department of Electrical Engineering, National Tsing Hua University, Hsinchu, Taiwan 30013

[4] Department of Engineering and System Science, National Tsing Hua University, Hsinchu, Taiwan 30013

[5] City University of Hong Kong, Department of Materials Science and Engineering, Tat Chee Avenue, Kowloon, Hong Kong 852

*Corresponding author email: rmho@mx.nthu.edu.tw / cclee@pme.nthu.edu.tw

$ Aum Sagar Panda, Cheng-Hsun Tung, and Jui-Chang Chuang contributed equally to this work.

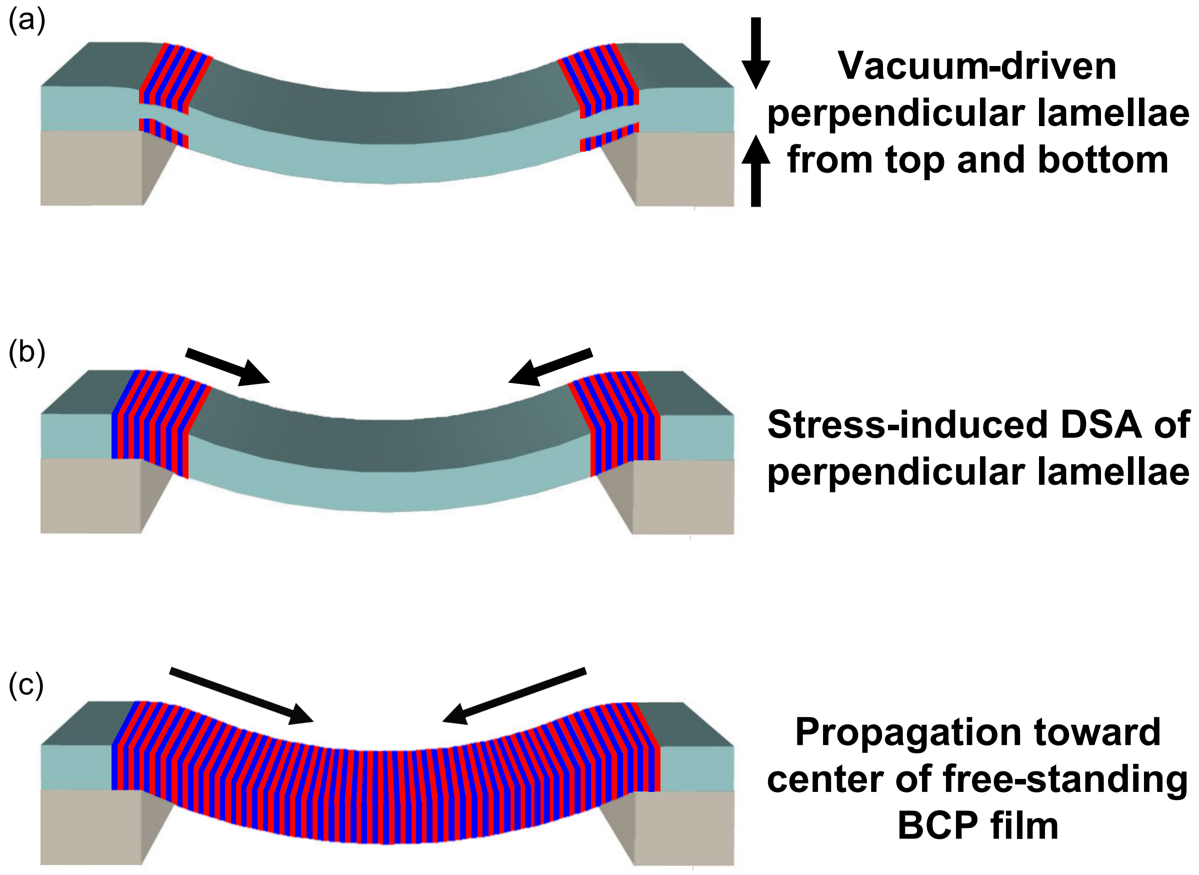

Figure S1. Schematic illustration of the proposed mechanism of stress-induced lamellar alignment. (a) Perpendicular lamellae nucleate initiated simultaneously from the top and bottom surfaces near the edge. (b) Edge-localized tensile stress promotes unidirectional alignment of the lamellar normal. (c) The aligned perpendicular lamellae propagate inward.

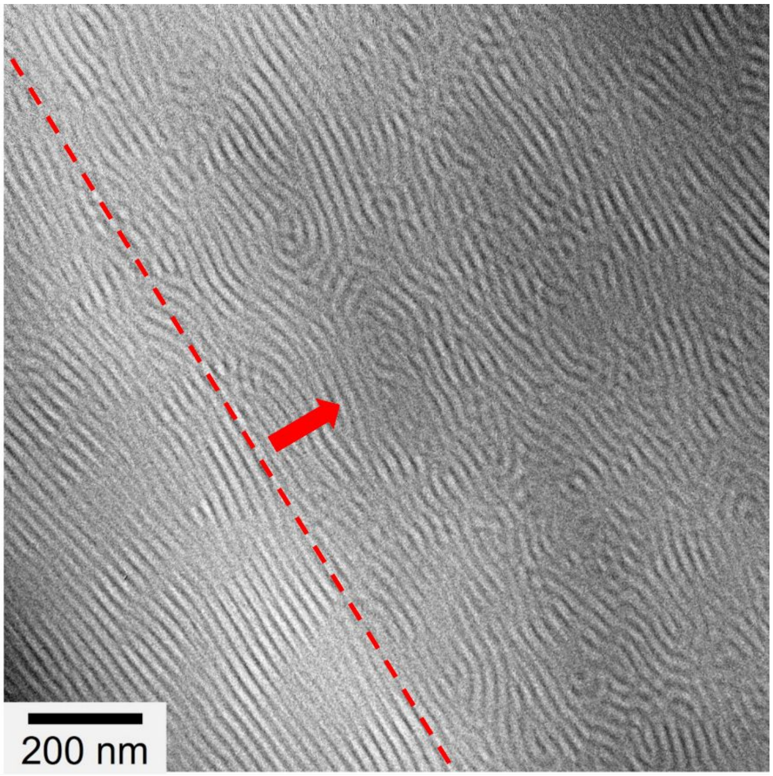

Figure S2. TEM micrograph of free-standing PS-*b*-PDMS thin film with self-assembled perpendicular lamellae away from the edge of TEM grid after thermal annealing at 300 ^{o}C under 10^{-4} Pa for 120 mins. The red arrow indicates the direction away from the supporting grid edge.

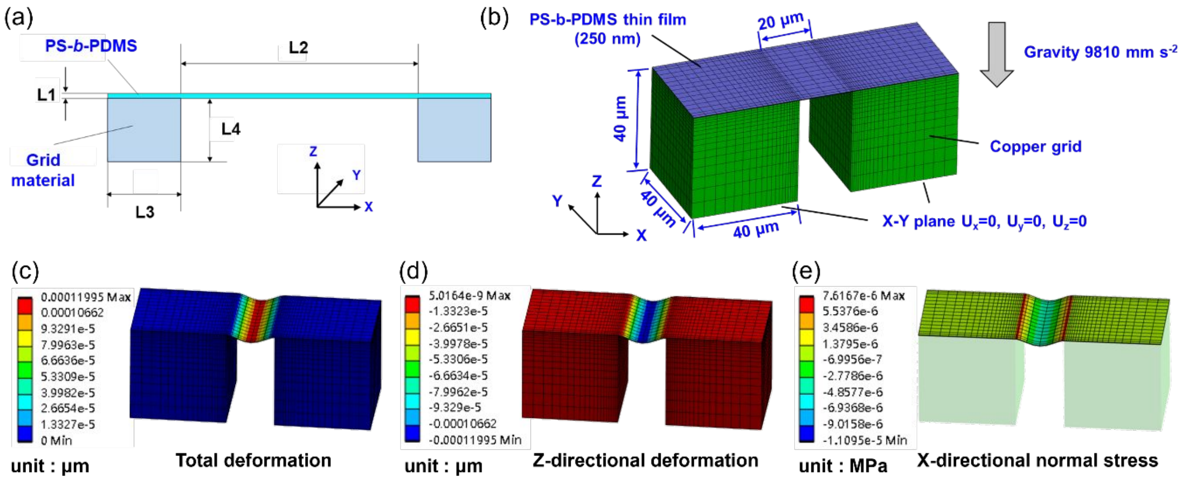

Figure S3. Stress analysis of PS-*b*-PDMS free-standing thin film on copper grid using FEA analysis. (a) Schematic illustration of the model geometry; L1 is fixed at 250 nm (film thickness of PS-*b*-PDMS), while L2–L4 are varied across different cases (see Table 1). (b) Schematic diagram of the FEA model geometry of the free-standing PS-*b*-PDMS thin film on copper grid. The PS-*b*-PDMS thin film is suspended over a square copper grid (40 µm pitch, 20 µm bar width), with gravity applied downward (9810 mm s^{-2}). The film is fixed at the boundary (X-Y plane) with zero displacement in all directions (Ux = Uy = Uz = 0). (c) Total deformation of the free-standing PS-*b*-PDMS thin film on copper grid. (d) Out-of-plane (Z-direction) displacement profile. Maximum deformation occurs in the central suspended region. (e) X-directional stress distribution of the free-standing PS-*b*-PDMS thin film. Tensile stress is localized along the edges of the grid bars.

Materials	No.	L1 (μm)	L2 (μm)	L3 (μm)	L4 (μm)
Copper grid	Set 1	0.25	20	40	40
SiO_2 substrate	Set 2	0.25	2	2	0.2
	Set 3	0.25	1	1	0.2
	Set 4	0.25	0.8	0.8	0.2
	Set 5	0.25	0.5	0.5	0.2
	Set 6	0.25	0.3	0.3	0.2

Table S1. Summary of geometric parameters used in the FEA model. The film thickness (L1) is fixed at 250 nm, while the space width (L2), line width (L3), and depth (L4) of the copper grid or SiO_2 substrate are varied across different simulation cases.

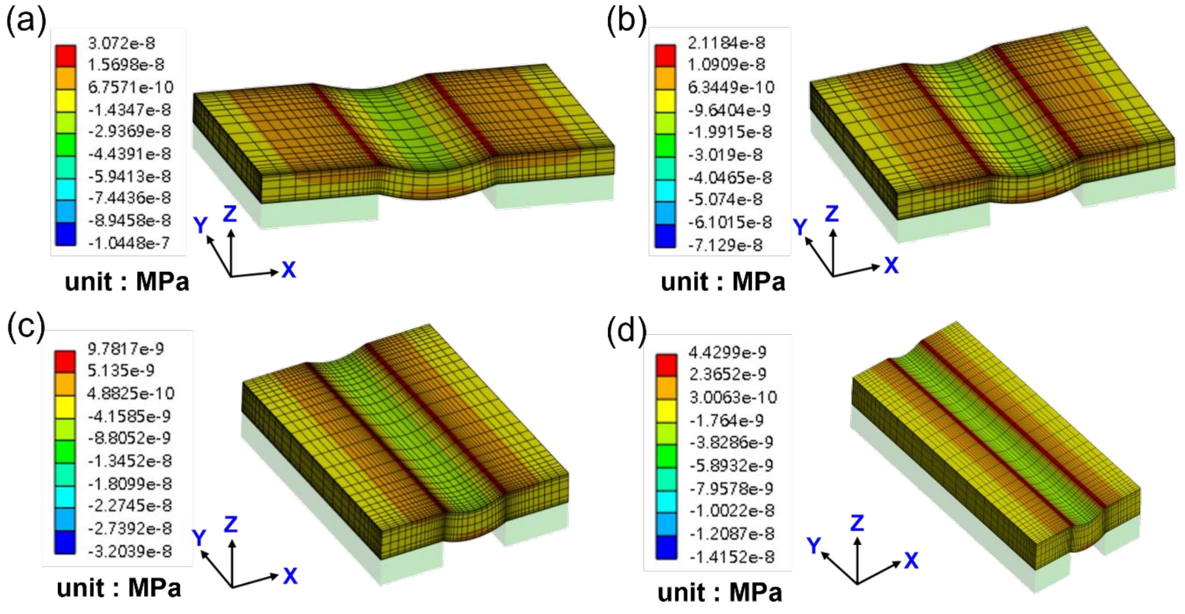

Figure S4. X-directional stress distribution of the free-standing PS-*b*-PDMS thin film on topographic SiO_2 substrates. Tensile stress is localized along the edges of the SiO_2 mesa with trench widths of (a) 1000; (b) 750; (c) 500; and (d) 300 nm.

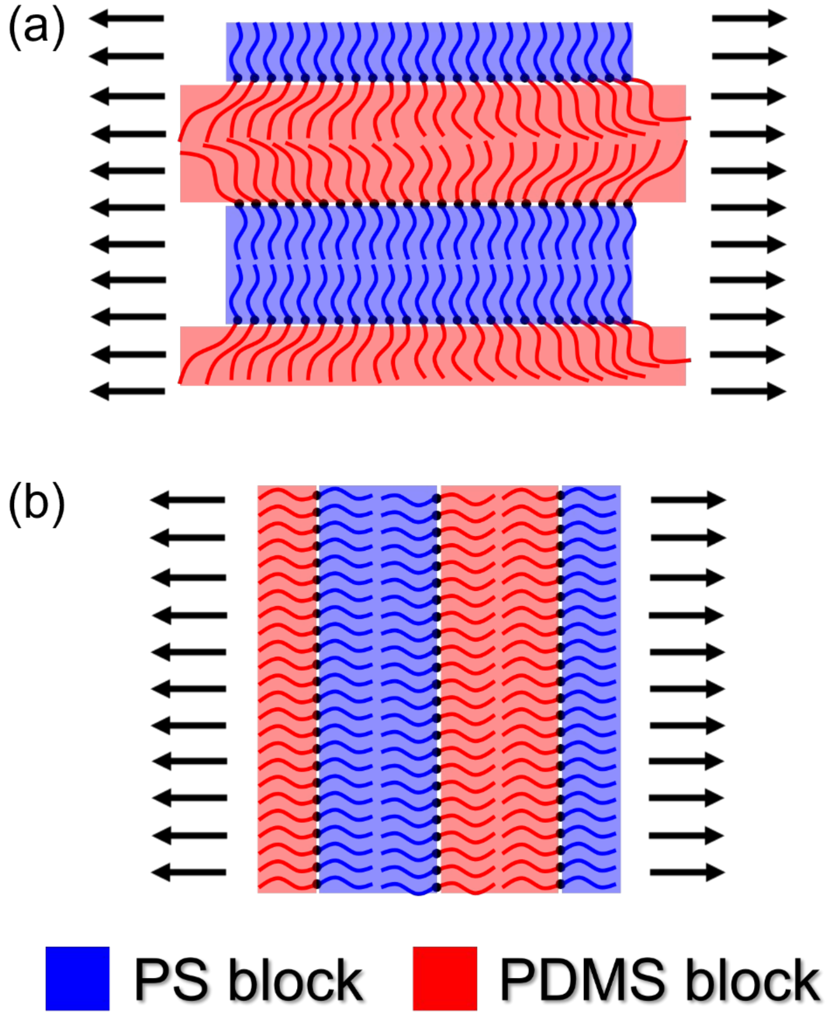

Figure S5. Schematic illustration of lamellae (a) perpendicular; (b) parallel to the edge of the copper grid. The black arrow indicates the direction of the stress experienced by the PS-*b*-PDMS thin film. The perpendicular arrangement in (a) is considered less favorable, as the elastic modulus discrepancy between PS and PDMS may lead to asymmetric deformation under stress. This asymmetry arises because the much softer PDMS undergoes greater tensile strain, resulting in chain frustration and hindering the formation of well-ordered lamellae.

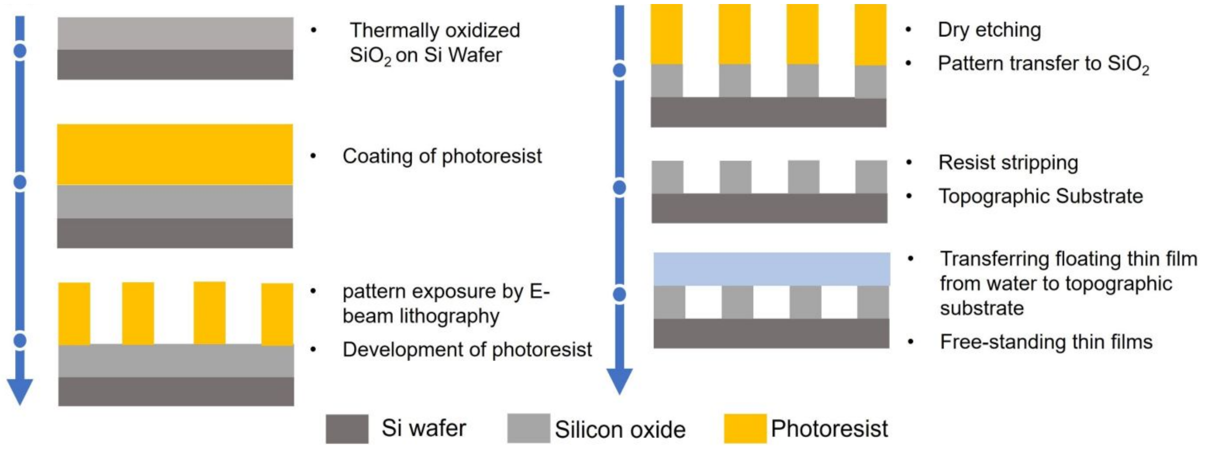

Figure S6. Schematic illustration for stepwise fabrication of trench pattern on a silicon wafer.

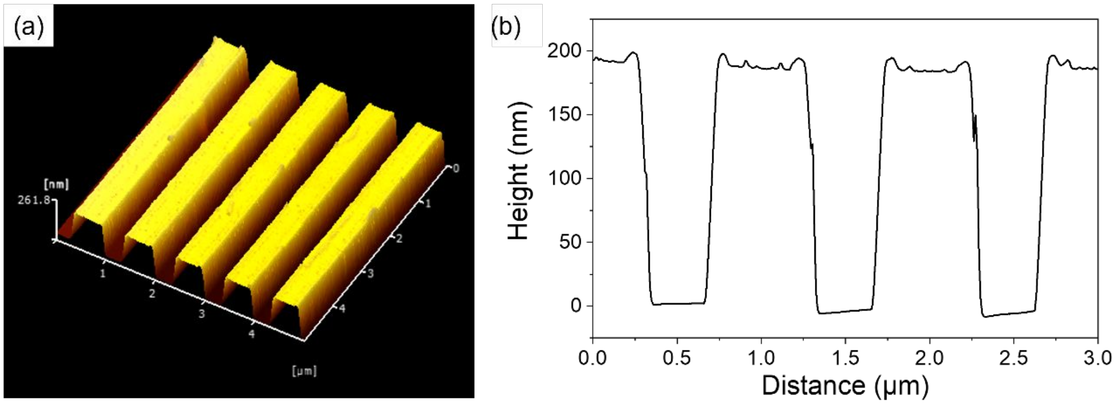

Figure S7. (a) The AFM 3D image of the topographic substrate with trench width of 500 nm fabricated by e-beam lithography; (b) Corresponding line profile extracted from top-view AFM image. The mesa and trench are defined with a 1:1 width ratio by e-beam lithography.

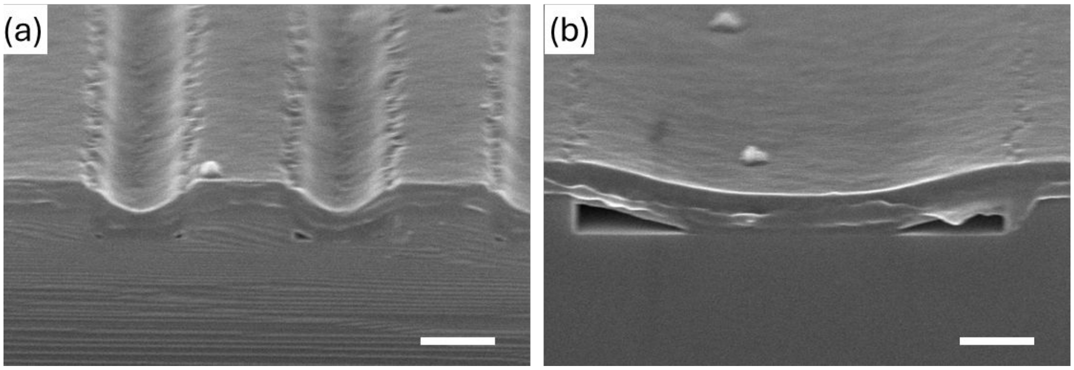

Figure S8. Cross-sectional SEM images of PS-*b*-PDMS thin films supported on the trench width of (a) 500 nm; (b) 3 μm after short-time (3 min) thermal annealing under vacuum.

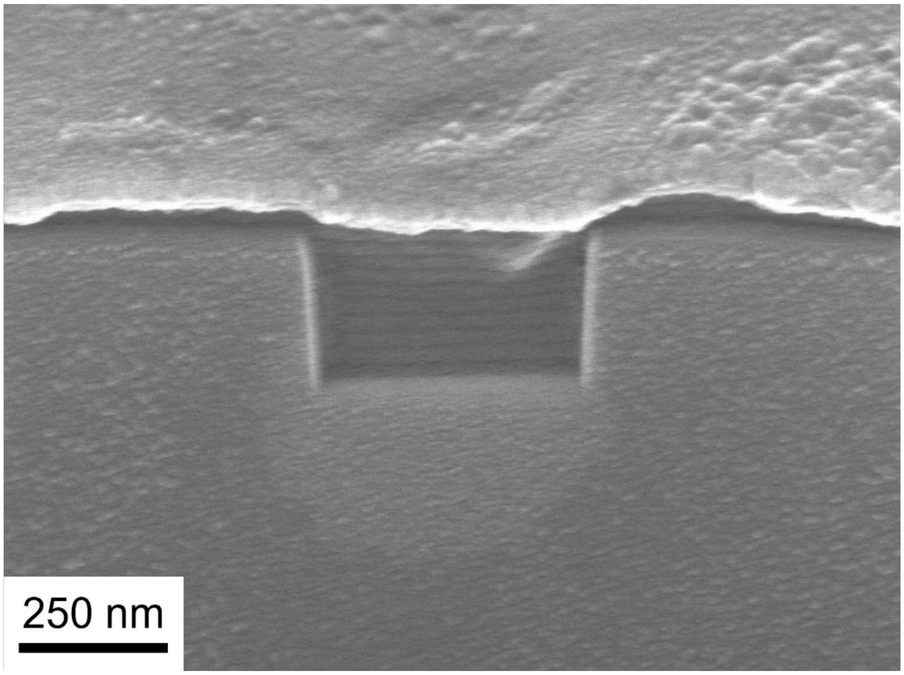

Figure S9. Cross-sectional FE-SEM image of parallel lamellae within the trench after thermal annealing under ambient pressure at 300 °C for 2 hours.
